# Christmas Festivities and COVID-19: A Foreseeable Risk to Anticipate

**DOI:** 10.3389/fpubh.2020.639647

**Published:** 2021-02-11

**Authors:** Stefania Boccia

**Affiliations:** ^1^Section of Hygiene and Public Health, Department of Life Sciences and Public Health, Università Cattolica del Sacro Cuore, Rome, Italy; ^2^Department of Woman and Child Health and Public Health - Public Health Area, Fondazione Policlinico Universitario A. Gemelli IRCCS, Rome, Italy

**Keywords:** COVID-19, holidays, SARS-CoV-2, pandemic, family gathering

With almost 75 million cases of coronavirus disease 2019 (COVID-19) and over 1,650 million related deaths worldwide, we are approaching the end of an unprecedented year (1). The certainties with which the population lived until a few months ago have suddenly been disrupted, and just as some European Countries were beginning to adopt more relaxing approaches in containing the spread of the severe acute respiratory syndrome coronavirus 2 (SARS-CoV-2), a second wave of COVID-19 forced most of them to return to containment measures. As Christmas festivities' celebrations are approaching, recommendations from experts and policy makers are abounding on the media, stressing the importance of avoiding any mass family gatherings during this period. A communication from the expert Dr. Anthony Fauci has warned about another surge in COVID cases in the US that could follow the Christmas period, alongside the post-Thanksgiving rise, which is still being currently tackled. This discussion is indeed relevant also for the majority of European Countries, including Italy, France, Spain, and Germany, where the debate about possibly lifting containment interventions adopted from October 2020 has been a burning issue for weeks.

What have we learned from SARS-CoV-2 in terms of household transmission and mass gatherings? Most SARS-CoV-2 infections are spreading due to airborne exposure to infected individuals (including pre-symptomatics, who account for 45–50% of positive subjects), situated within 2 m of distance. The transmission is particularly effective when speaking, shouting, singing, and breathing heavily during exercises within closed poorly ventilated spaces. The Center for Disease Control has recently updated its guidance by acknowledging the potential for airborne spread of SARS-CoV-2 beyond the droplets^1^. Instead, although the transmission through fomites (contaminated surfaces) has not been documented yet, it is still considered possible. Properly worn respiratory masks can reduce the respiratory virus shedding in exhaled breaths, thus they should be continued to be adopted in combination with physical distancing, hand hygiene, and adequate ventilation of indoor spaces. A recent report of the European Center for Disease Control recommends that heating, ventilation, and air-conditioning systems, if well-maintained and adapted for use during the COVID-19 pandemic, may have a complementary role in decreasing potential airborne transmission of SARS-CoV-2^2^. We also know that eating and drinking on-site at locations that offer such options might be important risk factors associated with SARS-CoV-2 transmission, mainly due to people removing their masks once they are seated^3^.

How do we continue to apply the preventive gold rules for COVID-19 during the coming season's celebrations that, in Europe, are expected to take place in closed spaces because of the winter season? Should we avoid any form of household and mass gatherings indoors and outdoors? These questions are currently at the center of the public debates in the context of the containment measures to undertake in the different EU Countries (https://www.nature.com/articles/d41586-020-03545-1). Familial transmission is responsible for around 70% of SARS-CoV-2 transmission when widespread community control measures are in place (2). We know that household secondary attack rate (SAR) is roughly 27%, which corresponds to a 10 times higher odds of SAR compared to others (2). In Wuhan, the reproduction number (R) dropped from 3.54 to 1.18 after lockdown and cordon sanitaire but reached 0.51 in 2 weeks when complete isolation of cases outside the home was implemented (3). Limiting the size of gatherings is a measure to reduce the likelihood of SARS-CoV-2 spreading to large number of people. A recent analysis reported that the highest reduction in the effective R is achieved when gatherings are limited to 10 people or less (36%; 16–53%) as opposed to 100 or less (21%; 1–39%) (4).

The duration for which people stay indoors is also associated with the attack rate, especially when it comes to community gatherings. For example, in March, during a 2.5 h indoor choir practice in Washington where no preventive measures were adopted, the attack rate was 85.2%^4^. Although in most of the EU Countries wearing masks is mandatory in indoor spaces, this aspect might be especially relevant in the context of holiday season celebrations in churches where singing is a common practice. Outdoor gathering events also represent a risky situation. A recent retrospective analysis of the change in COVID-19 incidence rate during the 2-weeks following outdoor mass gatherings in the US reported an average of 1.5-fold increase (5). In Italy, a large outdoor mass gathering during the UEFA Champions league football match of February 19, followed by extensive celebrations at a time where the first COVID-19 case was not yet detected, is supposed to have contributed to the 567% excess mortality documented in the Bergamo province (6).

With these considerations in mind, some general recommendations might be considered during the upcoming season celebrations in order to avoid the risk of COVID-19: household gatherings with non-cohabitants should be avoided, especially if elderly people are involved, or the number of participants should be limited according to the available space, to maintain proper physical distance; persons should wear masks and avoid eating at tables if this implies removing masks for prolonged periods of time; elderly people might be seated apart; spaces should be properly ventilated if deemed possible according to the outside temperatures; large outdoor mass gathering should be avoided. Figure 1 reports a map of the different containment measures adopted in the context of Christmas celebrations from selected European Countries and the UK on December 27, 2020.

**Figure 1:**
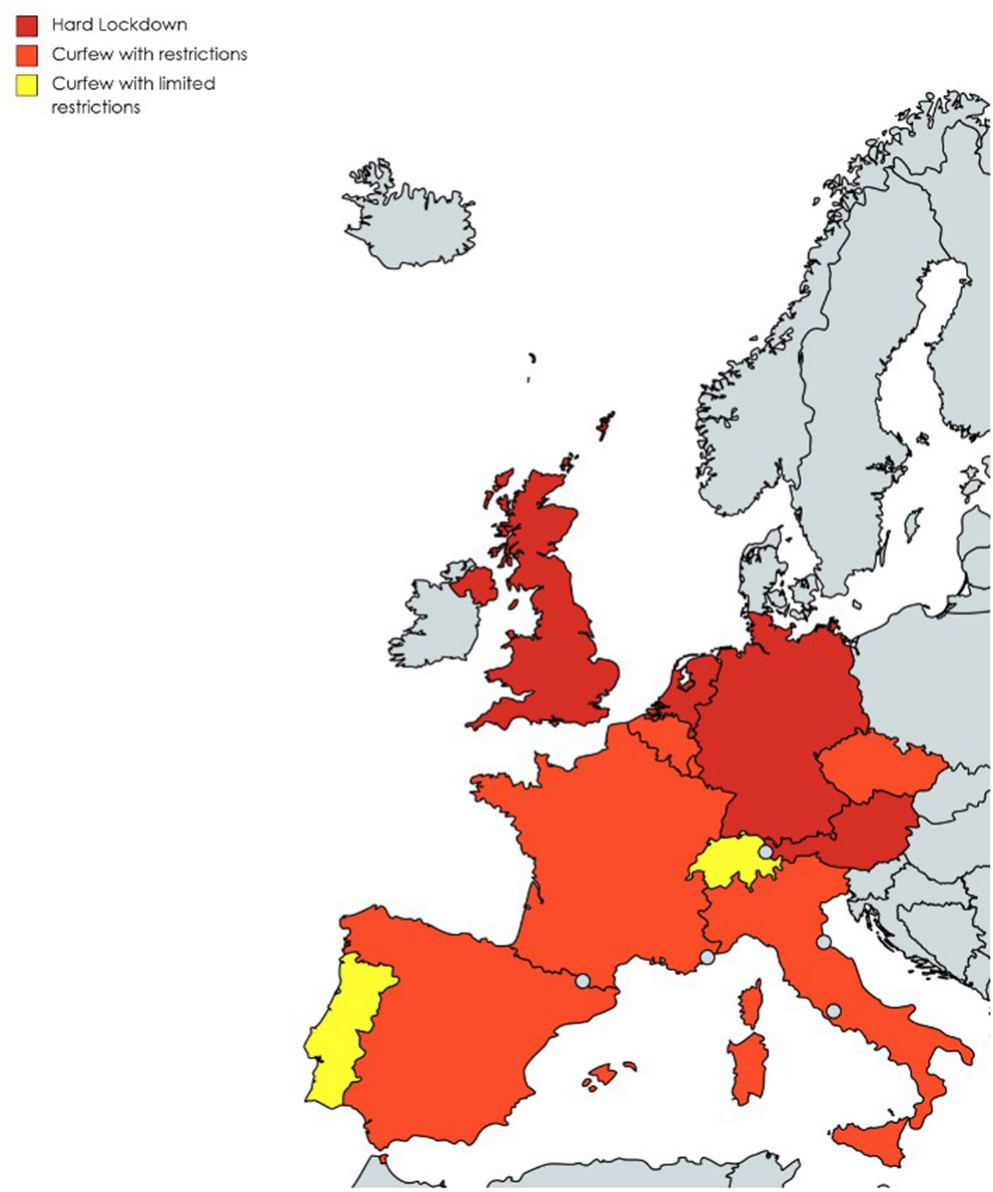
Containment measures in a selected number of the European countries and the UK during Christmas festivities (details according to the colored countries in the Appendix). **Austria, Germany, The Netherlands, United Kingdom:** hard lockdown, closure of non-essential shops, only close family members in the same home; **Italy:** curfew, closure of non-essential shops, no travel between regions, limited number of subjects in the same home, hard lockdown limited to selected days; **Spain:** curfew, closure of non-essential shops, limited travel between regions, limited number of subjects in the same home; **Belgium and Luxembourg: c**urfew, limited number of subjects in the same home; **France:** curfew, closure of non-essential shops, allowed travel between region, limited number of subjects in the same home; **Czech Republic:** schools and all non-essential shops closed, limits on assembly or movement; **Switzerland: e**arlier closure of non-essential shops and public places, bars and restaurants closed on Sundays, limited number of subjects in the same home; **Portugal:** curfew, no limit to subjects (mask required) in the same home for Christmas and travels between regions allowed. Outdoor gatherings limited to six subjects on New Year's eve and no travel between regions.

Although extensive mass vaccination against COVID-19 will start not earlier than mid-2021, we can reasonably assume that the coming season's celebrations will be the last one presenting the COVID-19 pandemic's challenges. In June, the European Commission presented a European strategy to accelerate the development, manufacturing, and deployment of effective and safe vaccines against COVID-19, and it is also committed to ensuring that everyone who needs a vaccine gets it, anywhere in the world and not only at home. As public health professionals, it is time to continue reinforcing the relevance of individual responsibility in containing COVID-19 pandemic also in the coming season' celebrations.

## Author Contributions

SB designed and wrote this opinion piece.

## Conflict of Interest

The author declares that the research was conducted in the absence of any commercial or financial relationships that could be construed as a potential conflict of interest.

## Footnotes

^1^Available online at: https://www.cdc.gov/coronavirus/2019-ncov/more/scientific-brief-sars-cov-2.html

^2^Available online at: https://www.ecdc.europa.eu/en/publications-data/heating-ventilation-air-conditioning-systems-covid-19

^3^Available online at: https://www.cdc.gov/mmwr/volumes/69/wr/mm6936a5.htm

^4^Available online at: https://www.cdc.gov/mmwr/volumes/69/wr/mm6919e6.htm.
